# Predicting Tumor Regrowth in Patients Undergoing Non-Operative Management after Total Neoadjuvant Therapy

**DOI:** 10.1007/s12029-025-01342-5

**Published:** 2025-11-03

**Authors:** Kamil Erozkan, Emily Simon, Emily Steinhagen, Lauren Henke, Meagan Costedio, Jennifer Eva Selfridge, Satish E. Viswanath, Ronald Charles

**Affiliations:** 1 Division of Colorectal Surgery, Department of Surgery, University Hospitals Cleveland Medical Center, Cleveland, OH USA; 2 Radiation Oncology, University Hospitals Cleveland Medical Center, Cleveland, OH USA; 3 Division of Colorectal Surgery, Department of Surgery, University Hospitals Ahuja Medical Center, Beachwood, OH USA; 4 Hematology and Oncology, University Hospitals Cleveland Medical Center, Cleveland, OH USA; 5 Department of Biomedical Engineering, Case Western Reserve University, Cleveland, OH USA; 6 Division of Colon & Rectal Surgery, University Hospitals Cleveland Medical Center, 11100 Euclid Ave, Cleveland, OH 44106 USA

**Keywords:** Rectal Cancer, Total Neoadjuvant Therapy, Clinical complete response, Tumor regrowth, Non-operative management, Total mesorectal excision

## Abstract

**Introduction:**

Total neoadjuvant treatment (TNT) has become the standard of care for locally advanced rectal cancer (LARC), leading to increased rates of complete clinical response and expanding the potential for organ preservation through non-operative management (NOM) protocols. Despite these advances, tumor regrowth remains a concern, necessitating vigilant surveillance to ensure early detection. However, adherence to surveillance protocols is often suboptimal, and the factors influencing tumor regrowth during NOM have not been well defined. This study aims to identify predictors of tumor regrowth in patients undergoing NOM after TNT.

**Method:**

We conducted a retrospective review of patients with LARC who completed TNT at a single institution between 2019 and 2024. Patients who achieved sustained complete clinical response (cCR) for at least 12 months, as well as those who experienced tumor regrowth following cCR, were included. Patients with suspected regrowth who subsequently underwent surgery and were found to have a pathologic complete response (pCR) were excluded. Univariate analyses were performed to compare demographic, histopathologic, biochemical, clinical, radiological, and treatment-related factors between patients who experienced tumor regrowth and those who did not. The primary objective of our study was to identify predictors of tumor regrowth.

**Results:**

Among 137 patients with LARC, 44 patients (32.1%) achieved cCR following completion of TNT. Of these, 10 patients experienced tumor regrowth and subsequently underwent surgery, with histopathology revealing a pCR in 2 cases. Currently, 11 patients remain in their first year of NOM, and 3 patients were lost to follow-up. In total, 20 patients sustained cCR. A total of 28 patients (25% female) with a mean age of 62.4 years (± 13) were included in the univariate analysis. No statistically significant differences were observed in demographic, histopathologic, biochemical, clinical, radiological, or treatment-related factors between patients who experienced tumor regrowth and those who did not (Table 1).

**Conclusion:**

This study did not identify any predictors of tumor regrowth in patients undergoing NOM after TNT. The limited number of events severely restricted the power to detect statistically meaningful associations. Nevertheless, this area warrants further investigation to better tailor surveillance strategies and optimize NOM recommendations.

## Introduction

Total neoadjuvant therapy (TNT) has emerged as a promising treatment strategy for select patients with locally advanced rectal cancer (LARC). Previously, standard management consisted of preoperative chemoradiotherapy, followed by total mesorectal excision (TME) and adjuvant chemotherapy. However, this approach leads to substantial rates of local and distant recurrence [1, 2]. TNT involves the administration of all systemic chemotherapy and radiation before surgery. This approach has several potential benefits, including enhanced treatment adherence, elimination of micrometastases, and increased rates of both clinical (cCR) and pathological complete response (pCR) [3, 4]. As a result, TNT facilitates organ preservation through non-operative management (NOM). This approach avoids the morbidity of surgery and the subsequent decreased quality of life related to postoperative bowel dysfunction or stomas [5].

Despite the promising oncological outcomes of TNT and the endorsement of the NOM approach by major guidelines, tumor regrowth is a significant concern, necessitating vigilant surveillance for its early detection. The reported tumor regrowth rates among patients undergoing TNT vary between 19% and 36% [6, 7]. However, adherence to surveillance protocols is frequently suboptimal [8], and the specific factors influencing tumor regrowth are not well defined. This study aims to identify predictors of tumor regrowth in patients undergoing NOM after TNT. We hypothesized that tumor-related factors are associated with tumor regrowth.

## Materials and Methods

### Study Design and Outcomes

This retrospective cohort study was conducted at a single tertiary care center. An IRB-approved database that included patients with rectal cancer between 2019 and 2024 was used. The study included patients diagnosed with LARC who were treated with TNT (defined as long-or short-course chemoradiation and either induction or consolidation chemotherapy), achieved cCR, and were selected for NOM. Patients who achieved sustained cCR for at least 12 months and those who experienced tumor regrowth following cCR during NOM were analyzed. Patients with suspected regrowth who subsequently underwent surgery and were found to have pCR were excluded.

## Treatment Algorithm and Definitions

LARC was defined as stage II (cT3/cT4 N0) or III (cN1/2) adenocarcinoma with a distal margin of ≤ 15 cm from the anal verge, as determined by colonoscopy and magnetic resonance imaging (MRI). Thoracic, abdominal, and pelvic computed tomography (CT) scans were performed to exclude the presence of distant metastases. All cases at our institution were reviewed by a multidisciplinary tumor board, and individualized treatment decisions regarding TNT were made collaboratively.

The individualized TNT protocol included either long-course chemoradiotherapy, consisting of 50–50.4 Gy of radiotherapy delivered in 25–28 fractions with concurrent capecitabine, or short-course radiation therapy (25 Gy in five fractions) with an induction or consolidation chemotherapy phase. The chemotherapy regimen typically involved systemic therapy administered as either FOLFOX (fluorouracil, leucovorin, and oxaliplatin; eight cycles) or CapeOx (capecitabine and oxaliplatin; six cycles), tailored to the patient’s clinical profile and treatment goals.

Approximately 4–6 weeks after completing TNT, patients underwent restaging with digital rectal examination (DRE), flexible sigmoidoscopy, pelvic MRI, and CT of the chest, abdomen, and pelvis. cCR was determined based on congruent findings from post-TNT sigmoidoscopy and MRI. Regarding visual inspection, cCR was defined by the absence of palpable or residual tumors, with permissible findings limited to mucosal whitening or an inflexible rectal wall with scarring observed on rectal examination and sigmoidoscopy. Regarding imaging findings, MRI diffusion sequences showing low signal intensity consistent with fibrosis and no tumor signal [magnetic resonance tumor regression grade (mrTRG 1)] or a fibrosis rate exceeding 75% with minimal tumor signal (mrTRG 2) were considered cCR. Patients who achieved cCR were offered NOM after review by the multidisciplinary tumor board.

Our institution’s active surveillance protocol for rectal cancer includes DRE, carcinoembryonic antigen (CEA) testing, and flexible sigmoidoscopy every 3 months for the first 2 years, every 6 months during years 3 and 4, and annually from years 5–10. Pelvic MRI is performed every 3 months for the first 4 years, followed by annual imaging. CT scans of the chest, abdomen, and pelvis are conducted every 6 months for the first 2 years and then annually. Colonoscopy is scheduled for 1 year post-treatment, with subsequent evaluations based on the findings. During the NOM surveillance period, any irregularities observed on endoscopy, such as residual superficial ulcers, palpable nodules, or mucosal polyps, are considered suspicious for regrowth. On surveillance imaging, mrTRG 3, 50 per cent tumor/fibrosis; mrTRG 4, less than 25 per cent fibrosis and predominant tumor signal; and mrTRG 5, no fibrosis are considered regrowth. In cases of tumor regrowth, TME is typically recommended following restaging and multidisciplinary tumor board review.

## Data Collection and Statistical Analysis

The collected variables included patient demographics (e.g., age at diagnosis, sex, race, ethnicity, body mass index [BMI]), baseline tumor characteristics (e.g., histologic grade, mismatch repair [MMR] status, tumor size, tumor distance from the anal verge, tumor location, extramural invasion [EMI], extramural vascular invasion [EMVI], mesorectal fascia [MRF] involvement, internal anal sphincter [IAS] involvement based on pre-treatment MRI), T and N stages as defined by the American Joint Committee on Cancer (AJCC), preoperative carcinoembryonic antigen (CEA) levels, history of preoperative diversion, and treatment pathway (e.g., treatment sequence, chemotherapy, and radiotherapy regimens). The study analyzed several key time intervals in the patient’s treatment, including the interval from diagnosis to initiation of the first TNT step, the duration of TNT from start to completion, and the time interval between the completion of TNT and restaging. The NOM period extended from the completion of the TNT to the latest follow-up.

Data analysis was conducted using Jamovi software (version 2.3.38). The Shapiro-Wilk test was used to assess the normality of the data distribution. Categorical variables are presented as frequencies (%), while continuous variables are reported as either mean (± standard deviation [SD]) or median (interquartile range [IQR]), depending on the distribution. Univariate analyses were used to compare demographic, histopathological, biochemical, clinical, radiological, and treatment-related factors between patients with and without tumor regrowth. Categorical variables were compared using the Chi-square or Fisher’s exact test, as appropriate, whereas numerical variables were compared using either the Mann-Whitney U test or Student’s t-test, based on normality. Statistical significance was set at *p* < 0.05.

## Results

**Fig. 1:**
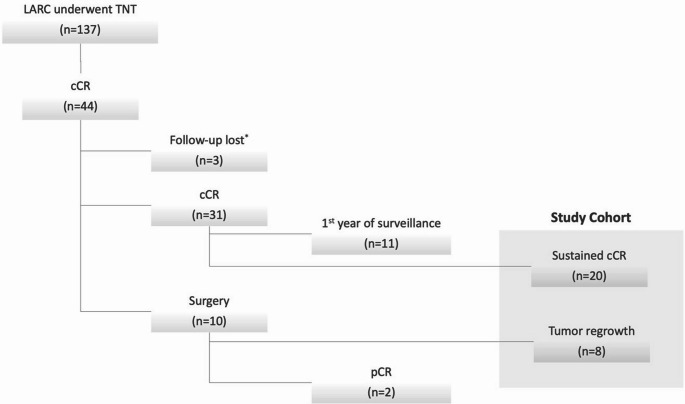
Diagram of study groups. TNT: Total neoadjuvant treatment, LARC: Locally advanced rectal cancer, cCR: clinical complete response, pCR: complete pathological response, * After the completion of TNT

A total of 137 patients diagnosed with LARC were included (Fig. 1). Among those who underwent TNT, 31 were unable to complete the entire treatment regimen. The overall rate of treatment-dose reduction was 22.6%. Notably, only nine patients required dose reduction prior to completing 50% of the planned chemotherapy cycles (two cycles or less for CapeOx, three cycles or less for FOLFOX). Following TNT, 44 patients (32.1%) achieved cCR. Among them, 10 patients experienced tumor regrowth during the surveillance period. Of the 10 who underwent surgery, 2 achieved pCR, leaving 8 patients who experienced regrowth. Currently, 11 patients are in their first year of NOM surveillance, and three have been lost to follow-up. Ultimately, 20 patients sustained their cCR over the NOM period after the first year, while eight experienced regrowth, yielding a study cohort of 28 patients for the univariate analysis.

Univariate analysis (Table 1) did not reveal any statistically significant differences between patients with sustained cCR and those with tumor regrowth across multiple demographic, histopathologic, biochemical, clinical, radiologic, and treatment-related factors. The mean age at diagnosis was 65.3 (± 12.5) years for patients with sustained cCR and 55.4 (± 12.2) years for those who experienced regrowth (*p* = 0.06). The cohort included 25% females in both groups. The racial distribution consisted predominantly of White patients (80% and 87.5% in the sustained cCR and regrowth groups, respectively), with no significant association between race or ethnicity and the likelihood of disease regrowth. The median BMI was 30.9 kg/m² (± 5.7) in the sustained cCR group and 29.1 kg/m² (± 5.3) in the regrowth group (*P* = 0.26).

**Table 1:** Univariate analysis between patients with sustained cCR and patients with regrowth

		*Sustained cCR (n=20)*	*Regrowth (n=8)*	Odds ratio (95% CI)	*p*-value
Age, year (±SD)		65.3 (12.5)	55.4 (12.2)	-	0.06
Female Sex, n (%)		5 (25%)	5 (25%)	1.00 (0.15-6.64)	1.00
Race, n (%)	Asian Black White	1 (5%) 3 (15%) 16 (80%)	0 (0%) 1 (12.5%) 7 (87.5%)	n/a	1.00
Ethnicity, n (%)	Non-Hispanic	20 (100%)	8 (100%)	n/a	-
BMI, kg/m^2^ (±SD)		30.9 (5.7)	29.1 (5.3)	-	0.26
Histologic Grade, n (%)	Well Moderately Poorly	3 (15%) 15 (70%) 3 (15%)	0 (0%) 8 (100%) 0 (0%)	n/a	0.45
MMR status, n (%)	Proficient Deficient Unknown	17 (85%) 1 (5%) 2 (10%)	7 (87.5%) 0 (0%) 1 (12.5%)	n/a	1.00
Gene mutation, n (%)		3 (15%)	3 (37.5%)	3.40 (0.51-22.4)	0.31
Tumor Size, cm (±SD)		4.3 (1.5)	4.2 (1.5)	-	0.87
Tumor Location, n (%)	Low Rectum Mid Rectum Upper Rectum	6 (30%) 8 (40%) 6 (30%)	2 (25%) 4 (50%) 2 (25%)	n/a	1.00
Distance to AV, cm (±SD)		8.1 (3.6)	7.4 (3.6)	-	0.66
Sphincter Invasion, n (%)		1 (5%)	0 (0%)	0.76 (0.02-20.7)	1.00
MRF Invasion, n (%)		6 (30%)	2 (25%)	0.77 (0.12-5.02)	1.00
EMI, n (%)		13 (65%)	7 (87.5%)	3.77 (0.38-37.1)	0.37
EMVI, n (%)		3 (15%)	2 (25%)	1.89 (0.25-14.2)	0.60
T, n (%)	T1 T2 T3 T4	0 (0%) 2 (10%) 11 (55%) 7 (35%)	0 (0%) 0 (0%) 7 (87.5%) 1 (12.5%)	n/a	0.31
N, n (%)	N0 N1 N2	8 (40%) 10 (50%) 2 (10%)	4 (50%) 3 (37.5%) 1 (12.5%)	n/a	0.84
Baseline CEA, ng/mL (IQR)		2 (1.5)	2.5 (2.2)	-	0.72
Preoperative diversion, n (%)		1 (5%)	1 (12.5%)	2.71 (0.14-49.5)	0.49
Treatment Sequence, n (%)	Induction – RT RT – Consolidation	2 (10%) 18 (90%)	0 (0%) 8 (100%)	2.30 (0.09-53.2)	1.00
Radiation Course	Long course Short course	14 (70%) 6 (30%)	5 (62.5%) 3 (37.5%)	1.40 (0.25-7.89)	1.00
Concurrent Chemotherapy, n (%)	5-Florouracil Capecitabine No	1 (5%) 13 (65%) 6 (30%)	0 (0%) 5 (62.5%) 3 (37.5%)	n/a	1.00
Induction/Consolidation Chemotherapy, n (%)	CapeOx FOLFOX Other	9 (45%) 7 (35%) 4 (20%)	5 (37.5%) 3 (62.5%) 0 (0%)	n/a	0.60
Chemotherapy completed, n (%)		16 (80%)	6 (75%)	0.75 (0.10-5.22)	1.00
Diagnosis–TNT initiation, day (±SD)		68.6 (47)	50.9 (22)	-	0.32
TNT duration, day (±SD)		176 (68)	157 (49)	-	0.47
Completion of TNT – Restaging, day (±SD)		44 (20)	40 (22)	-	0.70
WW time, month (±SD)		24.6 (12.3)	5.7 (1.5)	n/a	n/a

MMR: Mismatch repair, CEA: Carcinoembryonic antigen, AV: Anal verge, MRF: Mesorectal facia, EMVI: Extramural vascular invasion, EMI: Extramural Invasion, RT: Radiotherapy, TNT: Total neoadjuvant treatment

Regarding tumor characteristics, the histologic grade varied, with a higher percentage of patients with moderately differentiated tumors in the regrowth group (100%) than in the sustained cCR group (70%); however, this difference was not statistically significant (*p* = 0.45). Other tumor characteristics, including tumor size, distance from the anal verge, and MMR status, were not significantly associated with tumor regrowth. The mean tumor size was comparable between the groups (4.3 cm ± 1.5 for sustained cCR vs. 4.2 cm ± 1.5 for regrowth, *p* = 0.87). Additionally, the median distance to the anal verge was 8.1 cm (± 3.6) in the sustained cCR group and 7.4 cm (± 3.6) in the regrowth group (*p* = 0.66).

Extramural invasion (EMI) and extramural vascular invasion (EMVI) were more frequently observed in the regrowth group (87.5% and 25%, respectively) than in the sustained cCR group (65% and 15%, respectively), although these differences were not statistically significant (EMI *p* = 0.37, EMVI *p* = 0.60). Tumor staging at baseline, including T and N classifications, did not significantly predict tumor regrowth.

Regarding the sequence of treatments during TNT, most patients undergoing TNT received long-course chemoradiotherapy (67%, 50–50.4 Gy in 25–28 fractions) followed by consolidation chemotherapy. The type and sequence of chemotherapy and completion rates did not differ significantly between the sustained cCR and regrowth groups (*p* = 1.00, 0.60, and 1.00, respectively). Concurrent chemotherapy was primarily administered as capecitabine, with comparable administration rates between the groups (*p* = 1.00). The average duration of TNT was 176 (± 68) days for sustained cCR patients and 157 (± 49) days for those with regrowth (*p* = 0.47).

During surveillance, the sustained cCR group had an average observation period of 24.6 months (± 12.3), which was significantly longer than that of the regrowth group, with an average of 5.7 months (± 1.5). Among patients with tumor regrowth, seven underwent total mesorectal excision. Among these, one patient (12.5%) had a positive circumferential margin, while another (12.5%) had a near-complete total mesorectal excision. The proximal and distal resection margins were clear in all patients. Additionally, distant lung metastasis occurred in one patient (12.5%), who subsequently received chemotherapy.

## Discussion

Total neoadjuvant therapy (TNT) is increasingly recognized as the preferred approach for high-risk locally advanced rectal cancer, with studies showing higher response rates and improved treatment compliance compared with conventional chemoradiotherapy [3, 4]. Recent evidence also suggests improved oncological outcomes and greater potential for organ preservation with TNT [5, 9]. Reflecting this progress, the NCCN incorporated TNT into its 2022 guidelines for both CRM-threatened and CRM-negative tumors [10]. Although TNT offers advantages in terms of treatment response and watch-and-wait feasibility, tumor regrowth remains a significant concern and necessitates careful surveillance.

In this study, we analyzed 32% of patients with LARC in our cohort who underwent TNT, achieved cCR, and were selected for NOM. In this cohort, the regrowth rate was 23%, reflecting the significant challenge of pursuing NOM after initial cCR.

The findings of this study align with data from the International Watch & Wait Database, which reported a 2-year incidence of local regrowth of 25.2% in patients achieving cCR after TNT without surgery [11]. As younger patients in our cohort had a marginally higher incidence of regrowth, early onset LARC could be considered a potential predictor of regrowth, although this association did not reach statistical significance. Nonetheless, tumor regrowth remains a critical challenge, particularly in younger patients, as it may affect their long-term health outcomes and quality of life. Patients experiencing regrowth are known to be at an elevated risk of distant metastasis [12], and local management of regrowth is complex [13]. While all patients in our study had clear proximal and distal margins, one patient exhibited a positive circumferential margin and another had near-complete mesorectal excision, reflecting the complexity of the resection. Furthermore, one patient developed distant lung metastasis, indicating the potential for disease progression despite an initial cCR. These findings emphasize the importance of identifying regrowth predictors to refine patient selection for NOM and facilitate the early detection of local recurrence.

Based on existing studies, some potential predictors of tumor regrowth have been identified that were not replicated in our current study. For example, one previously identified risk factor for local regrowth is the total radiation dose, where patients who received less than 50.4 Gy have been reported to have a higher likelihood of developing local regrowth [12]. However, in our cohort, most patients underwent long-course radiation therapy, which may explain the lack of significant differences in regrowth observed between patients who received different radiation doses in our analysis. The InterCoRe consortium previously reported the risk of local regrowth to be approximately 20% for cT2, 30% for cT3, and nearly 40% for cT4 tumors, respectively [14]. In our cohort, most patients had cT3 disease; however, no statistical differences in local regrowth rates were observed between the T stages. In contrast, Habr-Gama et al. found that radiological nodal status was not associated with the risk of local regrowth or surgery-free survival in these patients [15]. Our findings align with this observation, as regrowth rates were comparable across the different N stages in patients with LARC. It is important to note that most of these potential predictors have been identified in patients who pursued NOM following chemoradiation alone rather than TNT. This distinction highlights the need for further research to evaluate predictors specifically in the context of TNT protocols.

Although no definitive predictors of regrowth have been identified following TNT, certain patterns have emerged. In the OPRA trial, tumor regrowth was observed in 44% and 29% of patients in the induction and consolidation chemotherapy arms, respectively [11]. Different chemotherapy regimens were used within these arms, leading to variations in the timing of chemotherapy completion, which in turn affected the intervals between chemotherapy completion and final treatment response assessment. Notably, an extended interval between the initiation of neoadjuvant therapy and surgery may have contributed to the increased risk of local failure. These findings suggest that treatment intervals and sequencing of chemotherapy (induction versus consolidation) may play a role in influence the likelihood of regrowth, highlighting the need for further studies to optimize treatment strategies. Similarly, findings from the RAPIDO trial, which compared TNT with consolidation chemotherapy versus standard preoperative nCRT, further highlighted the interplay between treatment timing and outcomes [3]. In RAPIDO, patients undergoing TNT had a longer interval between radiotherapy and surgery (22–24 weeks) than those in the standard nCRT arm (6–10 weeks). Although the pathological complete response (pCR) rate was higher in the TNT arm (28% vs. 14%; *p* < 0.0001), local failure was also more frequent in this group (12% vs. 8%; *p* = 0.07). This paradoxical result may reflect the repopulation of radio-chemoresistant clonogens during the extended waiting period, further emphasizing the need to refine predictive markers and optimize intervention strategies to minimize regrowth risks.

Identifying effective predictors of regrowth is complicated by the potential tumor biological changes induced by TNT or subsequent NOM. Residual tumor cells surviving chemoradiation may adapt by repairing damage from consolidation chemotherapy, selecting for more aggressive chemoresistant clones characterized by epithelial-to-mesenchymal transition [16]. Additionally, chemoradiation may promote local immune evasion in residual cells with specific genomic alterations, increasing the likelihood of both local regrowth and distant metastasis [17]. Any therapeutic benefit achieved through induction or consolidation chemotherapy may, in patients with cCR, inadvertently drive the selection and dissemination of small clusters of viable chemo-resistant tumor cells that may remain scattered within the area formerly occupied by the tumor [18]. This underscores the critical need to define robust predictors of regrowth to optimize patient selection and screening during NOM, which could potentially be enabled through recent advances in artificial intelligence and machine learning, which leverage routinely acquired imaging data [17].

This study has several limitations inherent to its design. First, the retrospective and single-center nature of the study may restrict the generalizability of our findings to broader patient populations. The most important limitation is the small sample size, which reduces the statistical power of the analyses and increases the risk of a type II error, potentially leading to false acceptance of the null hypothesis. Due to the limited sample size (*n* = 28), multivariable modeling could not be performed, which restricted our ability to control for potential confounders. This methodological limitation should be considered when interpreting these results. Consequently, the findings of this study should be regarded as exploratory and hypothesis-generating, rather than confirmatory. To more reliably identify predictors of tumor regrowth in patients undergoing NOM after TNT, larger multi-institutional and collaborative studies are essential to validate and strengthen these results. Although we evaluated the histologic grade, MMR status, and gene mutations, the inclusion of additional variables that better reflect tumor biology could enhance predictive analyses. Furthermore, variations in treatment sequencing and time intervalsstemming from the individualized TNT protocols at our centermay have influenced our results. Our relatively short follow-up period also posed a limitation, as 25% of the patients included in the NOM cohort were enrolled within the past year, potentially restricting the ability to assess long-term outcomes. Despite these limitations, our study is among the few that specifically investigates predictive factors for rectal tumor regrowth after TNT, emphasizing the clinical significance and utility of identifying such predictors to optimize patient selection and NOM.

## Conclusion

This study did not identify any significant predictors of tumor regrowth in patients undergoing NOM after TNT, which may be attributable to the limited sample size. However, the potential of early onset LARC as a predictor of regrowth requires further evaluation. This highlights the importance of investigating this subgroup more thoroughly to refine surveillance strategies and optimize recommendations for NOM, ultimately improving long-term outcomes, particularly in younger patients, whose quality of life may be more severely impacted by regrowth.

## Data Availability

The data that support the findings of this study are available on request from the corresponding author. The data are not publicly available due to privacy or ethical restrictions.
